# 

**Published:** 1895-09

**Authors:** 


					CONTENTS.
The Progress of Surgery in the last
Twenty-five Years.
By Horatio
Percy Symonds
161
On the Localisation of the Foramina
at the Base of the Skull.
By
Edward Fawcett, M.B., C.M. Edin.
173
the Diagnosis of Cancer and of
Simple Dilatation of the Stomach.
By J. Michell Clarke, M.A., M.D.
Cantab., M.R.C.P. Lond
181
The Administration of Ether.
By
John Freeman, F.R.C.S. Ed.
191
Progress of the Medical Sciences:
Medicine.
By R. Shingleton
Smith, M.D.
196
Surgery.
By James Swain, M.D.
202
Dermatology.
By Henry Waldo,
M.D.
207
Reviews of Books
214
Notes on Preparations for the Sick
231
The Library of the Bristol Medico-
Chirurgical Society "3*
The William F. Jenks Memorial Prize
237
Local Medical Notes
237
Scraps
239

				

